# Perceptions about the relative importance of patient care-related topics: a single institutional survey of its anesthesiologists, nurse anesthetists, and surgeons

**DOI:** 10.1186/s12871-016-0187-0

**Published:** 2016-03-22

**Authors:** Thomas R. Vetter, Joydip Barman, Arthur M. Boudreaux, Keith A. Jones

**Affiliations:** Department of Anesthesiology and Perioperative Medicine, University of Alabama School of Medicine, JT862, 619 19th Street South, Birmingham, AL 35249-6810 USA

**Keywords:** Surgical home, Perioperative care, Clinical care pathways, Healthcare outcomes, Patient-centered care

## Abstract

**Background:**

Persistently variable success has been experienced in locally translating even well-grounded national clinical practice guidelines, including in the perioperative setting. We have sought greater applicability and acceptance of clinical practice guidelines and protocols with our novel Perioperative Risk Optimization and Management Planning Tool (PROMPT™). This study was undertaken to survey our institutional perioperative clinicians regarding (a) their qualitative recommendations for (b) their quantitative perceptions of the relative importance of a series of clinical issues and patient medical conditions as potential topics for creating a PROMPT™.

**Methods:**

We applied a mixed methods research design that involved collecting, analyzing, and “mixing” both qualitative and quantitative methods and data in a single study to answer a research question. Survey One was *qualitative* in nature and asked the study participants to list as free text up to 12 patient medical conditions or clinical issues that they perceived to be high priority topics for development of a PROMPT™. Survey Two was *quantitative* in nature and asked the study participants to rate each of these 57 specific, pre-selected clinical issues and patient medical conditions on an 11-point Likert scale of perceived importance as a potential topic for a PROMPT™. The two electronic, online surveys were completed by participants who were recruited from the faculty in our Department of Anesthesiology and Perioperative Medicine and Department of Surgery, and the cohort of hospital-employed certified registered nurse anesthetists.

**Results:**

A total of 57 possible topics for a PROMPT™ was created and prioritized by our stakeholders. A strong correlation (*r* = 0.82, 95 % CI: 0.71, 0.89, *P* < 0.001) was observed between the *quantitative* clinician survey rating scores reported by the anesthesiologists/certified registered nurse anesthetists versus the surgeons. The quantitative survey displayed strong inter-rater reliability (ICC = 0.92, *P* < 0.001).

**Conclusions:**

Our qualitative clinician stakeholder survey generated a comprehensive roster of clinical issues and patient medical conditions. Our subsequent quantitative clinician stakeholder survey indicated that there is generally strong agreement among anesthesiologists/certified registered nurse anesthetists and surgeons about the relative importance of these clinical issues and patient medical conditions as potential topics for perioperative optimization and risk management.

**Electronic supplementary material:**

The online version of this article (doi:10.1186/s12871-016-0187-0) contains supplementary material, which is available to authorized users.

## Background

Persistently variable success has been experienced in locally translating even well-grounded national clinical practice guidelines, including in the perioperative setting [1, 2]. Recurrent barriers to health care providers adopting such guidelines include inadequate understanding, lack of agreement and perceived “real-world” practicality, clinicians’ concerns about loss of self-efficacy, low outcome expectations, and the inertia of existing practice [3]. Applicability can be enhanced by adapting clinical practice guidelines within the local context, and their acceptence can be improved by assessing barriers to their use [4].

**Fig. 1:**
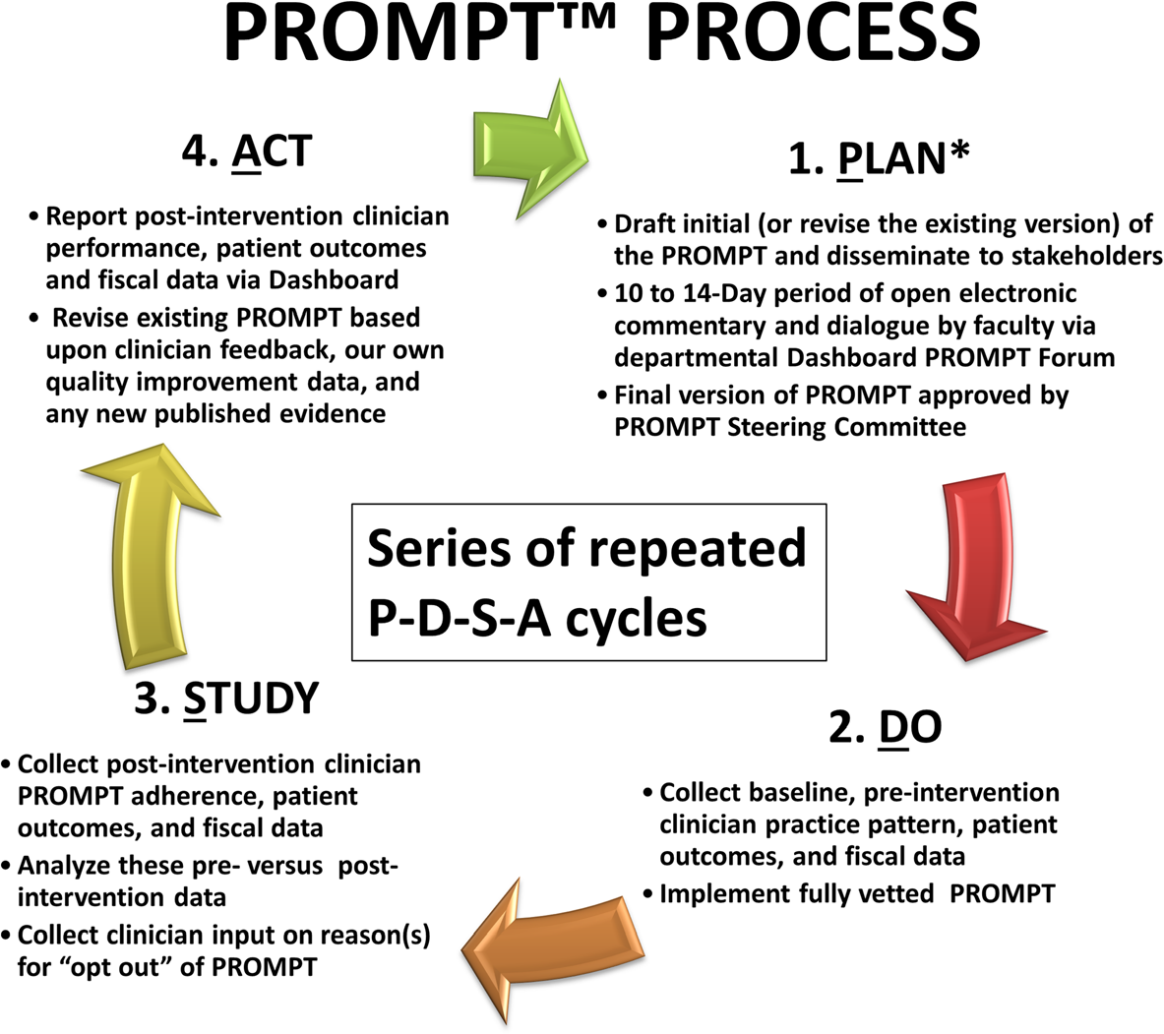
Iterative series of Plan-Do-Study-Act (PDSA) cycles applied to develop, implement, and revise a Perioperative Risk Optimization and Management Planning Tool (PROMPT™)

**Fig. 2:**
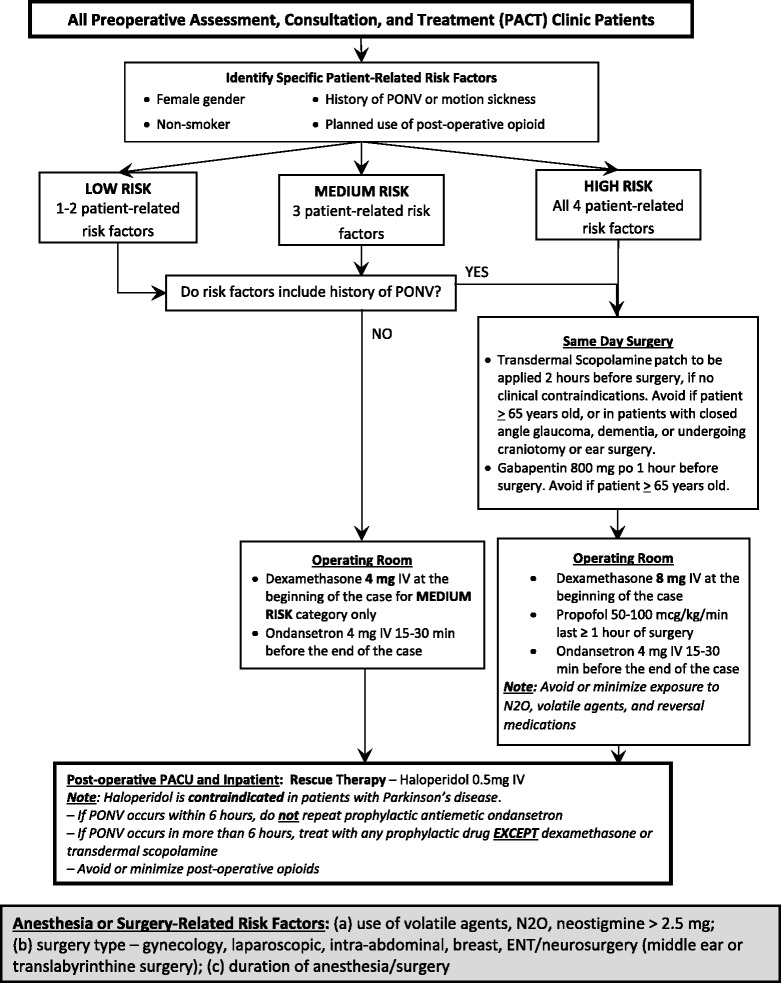
Prototypic Perioperative Risk Optimization and Management Planning Tool (PROMPT™) for the management of postoperative nausea and vomiting

At the University of Alabama at Birmingham (UAB), we have sought to achieve greater applicability and acceptance of clinical practice guidelines and protocols with our novel Perioperative Risk Optimization and Management Planning Tool (PROMPT™). A PROMPT™ is a merger of available published material with the equally valued expertise and consensus of institutional clinicians to arrive at current best practice. A PROMPT™ can serve as a real-time, front-line health informatics *decision support tool*, which can be applied by the health care team in their day-to-day operations and facilitate patient-specific, personalized care planning, decisions, and implementation. A PROMPT™ is not a static document but instead is essentially predicated on an iterative series of Plan-Do-Study-Act (PDSA) cycles that incorporate newly published data, concurrent institutional-level outcomes data, and local clinician feedback (Fig. 1). A prototypic PROMPT™ is the one we have developed at UAB for the management of postoperative nausea and vomiting (Fig. 2).

**Fig. 3:**
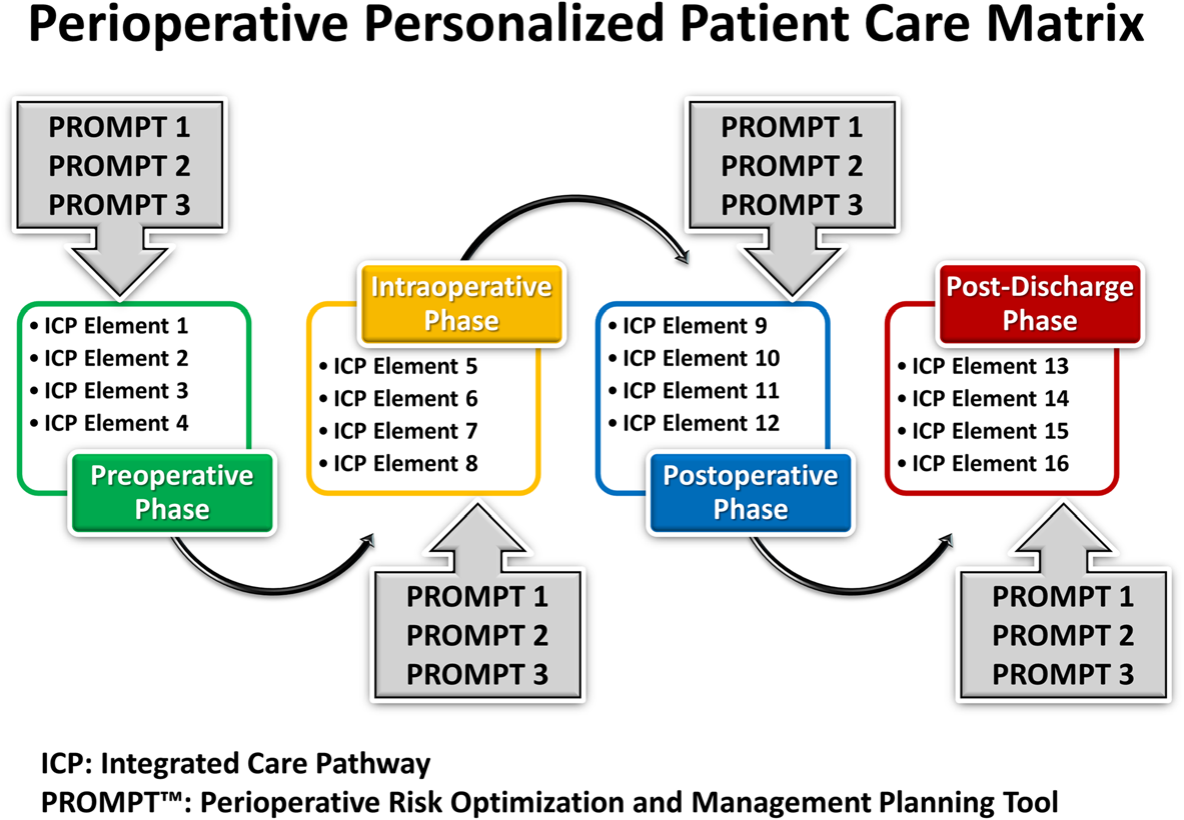
Perioperative Personalized Care Matrix comprised of both elements of an Integrated Care Pathway (ICP) and any applicable Perioperative Risk Optimization and Management Planning Tool (PROMPT™) [5]

While its efficacy has yet to be validly demonstrated, the PROMPT™ is intended to complement and to strengthen a surgical procedure-specific integrated care pathway. A patient-tailored “Perioperative Personalized Care Matrix” can be created by the amalgamation of all of the standardized elements of a surgical procedure-specific integrated care pathway and any applicable, patient condition-specific PROMPT™ (Fig. 3) [5]. This Perioperative Personalized Care Matrix is an integral component of our Perioperative Surgical Home model at UAB.

However, at the outset of our effort to develop, implement, and study our PROMPT™ concept, institutional stakeholder identification and prioritization of topics for a PROMPT™ were needed. A mixed methods research design involves collecting, analyzing, and “mixing” both qualitative and quantitative methods and data in a single study to answer a research question [6–8]. This mixed methods study was thus undertaken to survey our institutional perioperative clinicians regarding (a) their qualitative recommendations for (b) their quantitative perceptions of the relative importance of a series of clinical issues and patient medical conditions as potential topics for creating a PROMPT™.

## Methods

This continuous quality improvement (CQI) study was formally reviewed by the UAB Institutional Review Board (IRB) (E110311001). This study was approved under “Exempt” status [as defined by United States Department of Health and Human Services (45 CFR 46.101(b)(2)] by the UAB IRB, because the research involved the use of survey procedures in which the information obtained was recorded in such a manner that human subjects could not be identified, directly or through identifiers linked to the subjects. A waiver of signed written informed consent documentation was also granted by the UAB IRB. Per the UAB IRB, informed consent was instead obtained from all clinician study subjects prior to participation via their affirmative response on the initial page of the two online electronic surveys.

### Survey design

We administered two electronic, online clinician surveys.^1^ Survey One was *qualitative* in nature and asked the study participants to list as free text up to 12 patient medical conditions or clinical issues that they perceived to be high priority topics for development of a PROMPT™.

A “summative approach” to qualitative content analysis was then applied, in which the textual material was approached as single keywords, which were categorized based upon a review of the literature (i.e., conventional clinical sub-categories). This analysis of the patterns generated an interpretation of the contextual meaning of the specific terms (medical conditions or clinical issues) [9].

The free-text responses from this initial *qualitative* survey were hence grouped into the five categories of “Preoperative Testing,” “Medications and Technologies,” “Optimization of Co-Morbid Diseases,” “Perioperative Risk Reduction,” and “Provision of Comprehensive Care.” A final roster of 57 total possible topics for a PROMPT™ was created.

Survey Two was *quantitative* in nature and asked the study participants to rate each of these 57 specific, pre-selected clinical issues and patient medical conditions on a 11-point Likert scale [10, 11] of perceived importance as a potential topic for a PROMPT™. This 11-point Likert scale of perceived importance ranged from 0 = Not At All Important to 10 = Extremely Important. Once an importance rating score was electronically assigned to a given clinical issue or patient medical condition, the survey respondent could not return to that item.

### Survey validity and reliability testing

The two administered study surveys were questionnaires about clinician attitudes. The content and face validity of the two surveys were assessed and agreed upon by two experienced anesthesiologists (T.R.V. and K.A.J.). The inter-rater reliability of our *quantitative* clinician survey was assessed with an intraclass correlation coefficient (ICC) [12] between the importance scores for three generic topics, which were placed at the very beginning and at the very end of the list of the other 57 specific clinical issues and patient medical conditions. The individual clinicians’ paired ratings of the level of importance were significantly correlated for “hand washing in the operating room” (ICC = 0.93, *P* < 0.001), “patient hand-off communications” (ICC = 0.92, *P* < 0.001), and “first case on-time start rate” (ICC = 0.92, *P* < 0.001).

### Survey administration

Potential clinician study participants were recruited from all of the faculty members in the UAB School of Medicine Department of Anesthesiology and Perioperative Medicine and Department of Surgery, as well as the entire cohort of certified registered nurse anesthetists employed by UAB Hospital. A master list of anesthesiologists, surgeons, and certified registered nurse anesthetists was created using our institutional electronic directory.

These identified anesthesiologists, surgeons, and certified registered nurse anesthetists were then invited to participate in this study via an e-mail from the principal investigator (T.R.V.). The e-mail described the purpose of the study and provided the recipient with a hyperlink to the sequential online electronic *qualitative* and *quantitative* surveys located on surveymonkey.com. To maximize the survey response rate, two *qualitative* survey email invitations and subsequently three *quantitative* survey email invitations were sent at 10 day intervals to all potential study participants. The clinician survey responses were completely anonymous. The same participant pool was used for the *qualitative* survey and subsequent *quantitative* survey; however, because the clinician responses were anonymous, the degree of overlap could not be determined.

### Statistical methods

For the purposes of statistical analysis, because of our anesthesia care team approach at UAB, the *quantitative* survey responses from the anesthesiologists and certified registered nurse anesthetists were combined.

Continuous variables were reported using mean and standard deviation, or if the data were skewed, as a median and interquartile range. Categorical variables were reported using frequency counts and percentages. Parametric continuous demographic data were compared between groups using a *t*-test. Categorical demographic data were compared between groups using a Chi-square test or Fisher’s exact test. When indicated, a 95 % confidence interval was calculated for a point estimate.

A Pearson correlation coefficient was calculated and a scatterplot generated for the aggregated *quantitative* survey rating scores for each clinical issue and patient medical condition reported by the anesthesiologists/certified registered nurse anesthetists versus the surgeons.

The survey respondents’ self-reported *quantitative* clinician survey scores demonstrated a floor effect and ranged from 5.4 to 9.4 on the 11-point Likert scale of perceived importance. We established 20 % of this absolute score range of 4.0, equal to 0.8, to be a *minimally important score difference*. Of the 57 specific, pre-selected clinical issues and patient medical conditions, those were identified for which there was a mean difference of ≥ 0.8 between the anesthesiologists/certified registered nurse anesthetists and the surgeons. The aggregated *quantitative* clinician survey rating scores for each of these nine clinical issue and patient medical condition were compared between the anesthesiologists/certified registered nurse anesthetists versus the surgeons with an unpaired *t*-test.

Continuous data were assessed for normality with a Shapiro-Wilk test and by examining Q-Q plots, and if non-parametric, they were analyzed as such. No *a priori* sample size determination and power analysis was performed. For all univariate data analyses, a *P*-value of < 0.05 was considered significant. Statistical analyses were performed using SAS® Version 9.3 (SAS Institute Inc., Cary, NC).

## Results

### Demographics of clinician survey respondents

There were no statistically significant differences in the demographics of anesthesia care provider and surgeon survey groups (Table 1).

**Table 1:** Study participant demographics

	All Participants *N* = 83	Anesthesiologist *N* = 33	CRNA *N* = 15	Anesthesia Care Providers *N* = 48	Surgeons *N* = 35	Anesthesia Care Providers versus Surgeons	
						Standardized Difference Score	*P*-value
Age, mean ± SD	47.1 ± 9.8	45.6 ± 9.0	44.8 ± 10.6	45.4 ± 9.4	49.3 ± 10.0	0.40	0.086
Gender, N (%)						0.24	0.459
Female	17 (21)	5	6	11 (23)	6 (17)		
Male	64 (77)	26	9	35 (73)	29 (83)		
No response	2 (2)	2	0	2 (4)	0		
Experience, mean ± SD	14.3 ± 9.4	13.8 ± 9.3	12.5 ± 9.4	13.4 ± 9.2	15.5 ± 9.7	0.22	0.332
Race, N, (%)						0.21	0.466
Caucasian	67 (81)	27	13	40 (83)	27 (77)		
African American	3 (4)	1	1	2 (4)	1 (3)		
Hispanic	4 (5)	1	0	1 (2)	3 (9)		
Other	7 (8)	2	1	3 (6)	4 (11)		
No response	2 (2)	2	0	2 (4)	0		

*CRNA* certified registered nurse anesthetist

### Clinician qualitative survey responses

Of the 289 recruited anesthesiologists, certified registered nurse anesthetists, and surgeons, 64 (22 % response rate) completed the *qualitative* survey, between July 2014 and August 2014. Based upon the *qualitative* survey responses, a roster of 57 total clinical issues and patient medical conditions was created (Table 2).

**Table 2:** Perioperative Risk Optimization and Management Planning Tool (PROMPT™) qualitative survey results

A. Preoperative Testing	
1. Laboratory (11)	
2. Cardiovascular (10)	
3. Pulmonary function (2)	
B. Medications and Technologies	
1. Beta-blockers	
2. Calcium channel blockers	
3. Diuretics	
4. Angiotensin-converting enzyme inhibitors	
5. Angiotensin receptor blockers	
6. Anticoagulants (17)	
7. Coronary artery stents (6)	
8. Cardiac pacemakers (4)	
9. Implantable cardioverter defibrillators	
10. Insulin pumps	
11. Intrathecal pumps	
12. Other implantable devices (vagal nerve stimulators, deep brain stimulators)	
C. Optimization of Co-Morbid Diseases	
1. Arterial hypertension (25)	
2. Coronary artery disease (3)	
3. Cardiomyopathy	
4. Congestive heart failure (3)	
5. Cardiac arrhythmias (5)	
6. Diabetes mellitus (18)	
7. Cerebrovascular disease (2)	
8. Chronic obstructive pulmonary disease (2)	
9. Obstructive sleep apnea (10)	
10. Pulmonary hypertension	
11. Renal insufficiency (6)	
12. Cirrhosis and liver failure	
13. Anemia (8)	
14. Obesity (7)	
15. Infection and sepsis (11)	
16. Trauma (3)	
17. Chronic pain/chronic opioid use (15)	
18. Substance abuse (3)	
19. Malignant hyperthermia	
20. Reported penicillin allergy	
D. Perioperative Risk Reduction	
1. Cognitive delirium (7)	
2. Cognitive dysfunction (4)	
3. Nausea and vomiting (21)	
4. Deep venous thrombosis (8)	
5. Cerebrovascular accident	
6. Myocardial injury after non-cardiac surgery (“MINS”)	
7. Acute kidney injury and renal failure (2)	
E. Provision of Comprehensive Care	
1. Choice of anesthetic technique and agents (11)	
2. Airway management (4)	
3. Ventilation (12)	
4. Glucose management (not just in diabetics) (9)	
5. Nutrition (2)	
6. Fluid and Electrolyte Management (16)	
7. Patient-centered blood management (11)	
8. Patient medication instruction and compliance (5)	
9. Preoperative physical conditioning (“prehabilitation”) (2)	
10. Smoking cessation (3)	
11. Perioperative opioid sparring strategies (“multimodal analgesia”) (13)	
12. Perioperative sedative sparring strategies	
13. Preoperative antimicrobial prophylaxis and skin preparation	
14. Intraoperative hypothermia prevention (maintaining normothermia)	
15. Geriatric anesthetic and analgesic management (3)	

Note: Patient medical conditions or clinical issues identified by the survey participants were categorized in to five main sections (A through E above). Survey response counts are shows for each topic (if count > 1)

### Clinician quantitative survey responses

Of the 72 recruited anesthesiologists, 33 completed the *quantitative* survey (46 % response rate); of the 161 recruited surgeons, 35 completed the survey (22 % response rate); and of the 47 recruited certified registered nurse anesthetist, 15 completed the survey (32 % response rate), between September 2014 and October 2014. The aggregated *quantitative* clinician survey rating scores for all 57 clinical issues and patient medical conditions are reported in Table 3.

**Table 3:** Specific clinical issues and patient medical conditions sorted by descending order based on all participants’ mean importance score (0 = “Not At All Important,” 10 = “Extremely Important”)

Clinical Issue/Patient Medical Condition	All Participants	Anesthesia Care Providers	Surgeons
	(*N* = 83)	(*N* = 48)	(*N* = 35)
	Mean (95 % CI)	Mean (95 % CI)	Mean (95 % CI)
Coronary artery stents	9.2 (9.0, 9.4)	9.2 (9.0, 9.5)	9.2 (8.9, 9.6)
Airway management	9.1 (8.8, 9.5)	8.9 (8.4, 9.5)	9.4 (9.0, 9.8)
Myocardial infarction or injury after non-cardiac surgery	9.1 (8.8, 9.4)	9.2 (8.9, 9.5)	8.9 (8.4, 9.4)
Malignant hyperthermia	9.1 (8.7, 9.5)	9.2 (8.7, 9.7)	8.8 (8.1, 9.5)
Ventilation	9.1 (8.8, 9.4)	9.0 (8.5, 9.5)	9.2 (8.8, 9.6)
Implantable cardioverter defibrillators	8.9 (8.7, 9.2)	9.0 (8.7, 9.3)	8.8 (8.4, 9.2)
Pulmonary hypertension	8.9 (8.6, 9.2)	9.1 (8.8, 9.4)	8.5 (8.0, 9.0)
Congestive heart failure	8.8 (8.6, 9.1)	9.0 (8.6, 9.3)	8.6 (8.2, 9.0)
Cardiomyopathy	8.8 (8.5, 9.1)	8.9 (8.6, 9.3)	8.6 (8.2, 9.1)
Coronary artery disease	8.7 (8.5, 9.0)	8.8 (8.5, 9.2)	8.6 (8.1, 9.0)
Anticoagulants	8.7 (8.3, 9.1)	8.3 (7.8, 8.8)	9.3 (8.9, 9.7)
Cardiac arrhythmias	8.6 (8.4, 8.9)	8.7 (8.3, 9.0)	8.6 (8.2, 9.0)
Preoperative antimicrobial prophylaxis and skin preparation	8.6 (8.2, 9.0)	8.4 (7.8, 9.0)	9.0 (8.6, 9.4)
Cardiac pacemakers	8.6 (8.3, 8.9)	8.6 (8.2, 9.0)	8.6 (8.1, 9.1)
Acute kidney injury and renal failure	8.5 (8.2, 8.9)	8.8 (8.4, 9.2)	8.2 (7.6, 8.8)
Infection and sepsis	8.5 (8.2, 8.8)	8.4 (8.0, 8.8)	8.7 (8.2, 9.1)
Intraoperative hypothermia prevention (maintaining normothermia)	8.4 (8.1, 8.8)	8.6 (8.2, 9.0)	8.2 (7.5, 8.9)
Cardiovascular testing	8.3 (8.0, 8.6)	8.5 (8.1, 8.9)	8.0 (7.4, 8.5)
Cerebrovascular accident	8.2 (7.9, 8.6)	8.5 (8.1, 8.9)	7.8 (7.1, 8.5)
Patient medication instruction and compliance	8.2 (7.9, 8.5)	8.2 (7.8, 8.6)	8.1 (7.6, 8.7)
Laboratory testing	8.2 (7.8, 8.5)	8.3 (7.9, 8.7)	8.0 (7.4, 8.5)
Deep venous thrombosis	8.1 (7.8, 8.5)	7.9 (7.4, 8.5)	8.4 (8.0, 8.9)
Cerebrovascular disease	8.1 (7.8, 8.4)	8.4 (8.0, 8.7)	7.8 (7.2, 8.3)
Geriatric anesthetic and analgesic management	8.1 (7.8, 8.4)	8.2 (7.7, 8.7)	7.9 (7.5, 8.4)
Patient-centered blood management	8.0 (7.7, 8.4)	8.3 (7.8, 8.7)	7.7 (7.2, 8.2)
Trauma	8 .0 (7.6, 8.5)	8.5 (7.9, 9.0)	7.3 (6.6, 8.1)
Arterial hypertension	8.0 (7.7, 8.3)	8.1 (7.6, 8.6)	7.9 (7.4, 8.3)
Cirrhosis and liver failure	8.0 (7.6, 8.4)	7.9 (7.4, 8.3)	8.2 (7.6, 8.8)
Perioperative opioid sparing strategies (“multimodal analgesia”)	7.9 (7.5, 8.3)	8.2 (7.6, 8.7)	7.5 (7.0, 8.0)
Fluid and electrolyte management	7.8 (7.4, 8.2)	7.7 (7.1, 8.2)	8.0 (7.5, 8.5)
Beta-blockers	7.8 (7.4, 8.2)	7.6 (7.1, 8.1)	8.0 (7.4, 8.6)
Insulin pumps	7.8 (7.4, 8.2)	7.9 (7.4, 8.4)	7.6 (6.8, 8.4)
Obstructive sleep apnea	7.8 (7.4, 8.1)	7.8 (7.3, 8.3)	7.7 (7.3, 8.2)
Diabetes mellitus	7.7 (7.4, 8.1)	7.9 (7.4, 8.4)	7.5 (6.9, 8.1)
Chronic obstructive pulmonary disease	7.7 (7.4, 8.1)	7.7 (7.3, 8.1)	7.9 (7.3, 8.4)
Renal insufficiency	7.5 (7.1, 7.9)	7.4 (6.9, 8.0)	7.6 (6.9, 8.3)
Glucose management (not just in diabetics)	7.5 (7.1, 7.8)	7.5 (7.0, 8.0)	7.5 (6.9, 8.0)
Nausea and vomiting	7.4 (7.0, 7.8)	7.8 (7.3, 8.4)	6.8 (6.2, 7.4)
Choice of anesthetic technique and agents	7.4 (6.9, 7.9)	7.3 (6.6, 8.0)	7.5 (6.8, 8.3)
Preoperative physical conditioning (“prehabilitation”)	7.4 (7.0, 7.8)	7.6 (7.2, 8.1)	7.0 (6.3, 7.7)
Cognitive delirium	7.3 (6.8, 7.8)	7.6 (7.1, 8.2)	6.8 (6.1, 7.5)
Anemia	7.3 (6.9, 7.7)	7.3 (6.7, 7.8)	7.3 (6.8, 7.8)
Cognitive dysfunction	7.3 (6.8, 7.7)	7.6 (7.0, 8.2)	6.7 (6.1, 7.3)
Perioperative sedative sparing strategies	7.2 (6.7, 7.7)	7.5 (6.9, 8.1)	6.7 (6.1, 7.4)
Smoking cessation	7.0 (6.5, 7.5)	6.8 (6.1, 7.5)	7.2 (6.5, 7.9)
Chronic pain/chronic opioid use	6.9 (6.5, 7.3)	7.2 (6.6, 7.7)	6.6 (6.1, 7.1)
Substance abuse	6.9 (6.6, 7.3)	6.9 (6.4, 7.3)	7.0 (6.4, 7.7)
Obesity	6.9 (6.4, 7.3)	6.7 (6.1, 7.4)	7.1 (6.5, 7.7)
Other implantable devices (vagal nerve stimulators, deep brain stimulators)	6.9 (6.4, 7.3)	7.0 (6.3, 7.7)	6.7 (6.0, 7.4)
Nutrition	6.8 (6.4, 7.2)	6.4 (5.9, 7.0)	7.4 (6.9, 7.9)
Angiotensin receptor blockers	6.8 (6.4, 7.2)	7.2 (6.7, 7.7)	6.2 (5.6, 6.8)
Angiotensin-converting enzyme inhibitors	6.8 (6.4, 7.1)	7.1 (6.6, 7.6)	6.2 (5.6, 6.8)
Reported penicillin allergy	6.7 (6.2, 7.2)	6.9 (6.2, 7.5)	6.4 (5.6, 7.2)
Intrathecal pumps	6.7 (6.2, 7.2)	6.8 (6.2, 7.4)	6.6 (5.7, 7.4)
Calcium channel blockers	6.5 (6.2, 6.9)	6.5 (6.0, 7.1)	6.6 (6.0, 7.2)
Pulmonary function testing	6.0 (5.4, 6.6)	5.4 (4.6, 6.3)	6.8 (6.1, 7.4)
Diuretics	5.8 (5.4, 6.3)	5.6 (5.0, 6.2)	6.1 (5.4, 6.8)

**Fig. 4:**
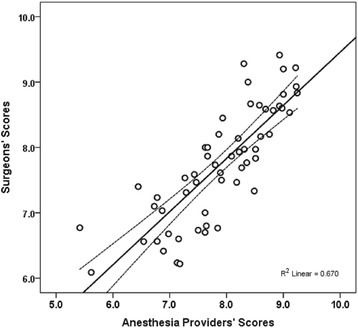
Scatterplot observed between the *quantitative* clinician survey rating scores reported by the anesthesiologists/certified registered nurse anesthetists versus the surgeons (*r* = 0.82, 95 % CI: 0.71, 0.89, *P* < 0.001)

A strong correlation (*r* = 0.82, 95 % CI: 0.71, 0.89, *P* < 0.001) was observed between the *quantitative* clinician survey rating scores reported by the anesthesiologists/certified registered nurse anesthetists versus the surgeons (Fig. 4). However, the scores for nine specific clinical issues and patient medical conditions met the minimally important score difference of 0.8. The scores for eight of these specific clinical issues and patient medical conditions were also statistically significantly different between the anesthesiologists/certified registered nurse anesthetists versus the surgeons. These nine items are rank-ordered by standardized difference score in Table 4.

**Table 4:** Specific clinical issues and patient medical conditions significantly differently rated by the anesthesia care providers (anesthesiologists/certified registered nurse anesthetists) versus the surgeons (0 = “Not At All Important,” 10 = “Extremely Important”)

Patient Condition	Anesthesia Care Providers (*N* = 48)	Surgeons (*N* = 35)	Standardized Difference Score	*P*-value
	Mean (95 % CI)	Mean (95 % CI)		
Anticoagulants	8.3 (7.8, 8.8)	9.3 (8.9, 9.7)	0.65	0.008
Trauma	8.5 (7.9, 9.0)	7.3 (6.6, 8.1)	0.62	0.011
Angiotensin receptor blockers	7.2 (6.7, 7.7)	6.2 (5.6, 6.8)	0.57	0.013
Nausea and vomiting	7.8 (7.3, 8.4)	6.8 (6.2, 7.4)	0.57	0.019
Nutrition	6.4 (5.9, 7.0)	7.4 (6.9, 7.9)	0.57	0.022
Pulmonary function testing	5.4 (4.6, 6.3)	6.8 (6.1, 7.4)	0.56	0.018
Angiotensin-converting enzyme inhibitors	7.1 (6.6, 7.6)	6.2 (5.6, 6.8)	0.51	0.025
Cognitive dysfunction	7.6 (7.0, 8.2)	6.7 (6.1, 7.3)	0.48	0.047
Cognitive delirium	7.6 (7.1, 8.2)	6.8 (6.1, 7.5)	0.40	0.094

## Discussion

This mixed methods study was undertaken to survey our institutional perioperative clinicians regarding (a) their qualitative recommendations for (b) their quantitative perceptions of the relative importance of a series of clinical issues and patient medical conditions as potential topics for creating a PROMPT™. Our qualitative data generated a comprehensive roster of 57 clinical issues and patient medical conditions. Our quantitative results indicate that there is generally strong agreement among anesthesiologists/ certified registered nurse anesthetists and surgeons about the relative importance of these clinical issues and patient medical conditions as potential topics for perioperative optimization and risk management. The nine clinical issues and patient medical conditions, for which there were significantly divergent prioritization, likely represent topics for which further efforts at achieving stakeholder consensus are indicated.

In our prior experience with instituting a standardized protocol for eye care management during anesthesia to prevent corneal injury, there was a significant initial reluctance among practitioners to comply with the proposed protocol [13]. Practice change did not fully occur and become part of our culture until outcomes data, showing efficacy, were repeatedly provided to practitioners. Lack of input from the involved practitioners in the development of this earlier protocol was thought to be a reason for their initial resistance to change practice.

Our present approach to continuous quality improvement thus began with “Start with *what*.” With our PROMPT™ concept, we first sought input from practitioners to determine what they perceived to be the most important topics in the care of our patients. This prioritization can now be used as a reason why a particular best practice change was selected—thereby hopefully increasing initial buy-in (i.e., “Start with Why”) [14]. This will include identifying and capitalizing upon crucial clinician motivators for performance improvement.

Nevertheless, with introduction of the PROMPT™ concept and a PROMPT™ focused on postoperative nausea and vomiting (Fig. 2), we have encountered initial attending physician pushback, mainly an unwillingness to practice “cookbook medicine.” However, a PROMPT™ is not prescriptive, “cookbook medicine” but instead serves as a best practice-based decision support tool. The successful development and implementation of a PROMPT™ requires not only robust informatics, analytics and decision support but also sustained robust grassroots clinician input and “buy-in.”

In addition to clinicians’ perceived importance of a given clinical issue or patient medical condition, other factors should be considered when prioritizing PROMPT™ topics for development and implementation. For example, we have observed that when the level of evidence to support a given PROMPT™ includes robust randomized clinical trials, systematic reviews, and meta-analyses, the degree of initial clinician buy-in can be more substantial. The prioritization of PROMPT™ topics may also be modified by practical matters, including the required resources, the complexity of the PROMPT™, and local institutional political considerations.

### Limitations

One weakness of our study was the relatively low overall qualitative survey response rate (22 %) and overall quantitative survey response rate (30 %), potentially undermining the validity of our findings due to non-response bias. It is likely that this minority of survey respondents were more motivated to describe the issues that they felt were important. Only their identified set of issues was in turn scored in the second phase of the study. However, non-response bias may be less of a concern in physician surveys than in surveys of the general public [15]. Likewise, higher physician survey response rates have not been associated with lower response bias [16].

Based on Rogers’ Diffusion of Innovation Theory [17], it is likely that our minority survey respondents are highly engaged Innovators and Early Adopters, and the majority non-respondents are Early Majority, Late Majority, and Laggards. Such early adopters could serve as “thought leaders” who subsequently increase buy-in from the whole group.

Our study might have been strengthened if we had more clearly specified that the clinician survey respondents rank the 57 items (clinical issues and patient medical conditions) from their own provider-perspective rather than from the patient perspective or the institutional/financial perspective.

The results of our clinician survey nevertheless may effectively serve as the impetus for achieving greater local stakeholder consensus on how to prioritize a series of clinical issues and patient medical conditions as topics for a PROMPT™.

## Conclusions

Our qualitative clinician stakeholder survey generated a comprehensive roster of clinical issues and patient medical conditions. The results of our subsequent quantitative clinician stakeholder survey indicate that there is generally strong agreement among anesthesiologists/certified registered nurse anesthetists and surgeons about the relative importance of these clinical issues and patient medical conditions as potential topics for perioperative optimization and risk management. We intend to undertake a future research to demonstrate if rank ordering problems department-wide and working on them together increases compliance with a specific PROMPT™.

## Additional files

Additional file 1: Perioperative Risk Optimization and Management Planning Tool (PROMPT™) Qualitative Clinician Survey. (PDF 65 kb)

Additional file 2: Perioperative Risk Optimization and Management Planning Tool (PROMPT™) Quantitative Clinician Survey. (PDF 73 kb)

## Abbreviations

CQI: continuous quality improvement
ICC: intraclass correlation coefficient
PROMPT: Perioperative Risk Optimization and Management Planning Tool
PSH: perioperative surgical home

## Footnotes

1 The two administered surveys are available in Additional file 1 and Additional file 2.
