# Supplementary material for: Perceptions about the relative importance of patient care-related topics: a single institutional survey of its anesthesiologists, nurse anesthetists, and surgeons
Source: BMC Anesthesiol. 2016 Mar 22;16:19. doi: 10.1186/s12871-016-0187-0 (PMC4804473; doi:10.1186/s12871-016-0187-0)
Supplement: Supplementary file 1 — Perioperative Risk Optimization and Management Planning Tool (PROMPT™) Qualitative Clinician Survey. (PDF 65 kb) [file 12871_2016_187_MOESM1_ESM.pdf]

Surveying the Clinical Practice Patterns within
the UAB Department of Anesthesiology

Qualitative PROMPT Survey – STUDY SURVEY ONE

Please answer the following questions:

What type of clinician are you? ☐ Anesthesiologist ☐ Surgeon ☐ Nurse Anesthetist

How many years after completing your training have you been in clinical practice? ______ years

How old are you? ______ years

What is your gender? ☐ Female ☐ Male

What is your race? ☐ African-American ☐ Caucasian ☐ Hispanic ☐ Other

A Perioperative Risk Optimization and Management Planning Tool (PROMPT) is a local clinician-designed approach to promoting standardization of care, which accommodates patients' individual differences, respects care providers' clinical acumen, and keeps pace with the rapid growth of medical knowledge.

Please list up to 12 clinical care issues or medical conditions in surgical patients, which you feel are the most important topics for the development and implementation of a PROMPT here at UAB.

1. ______________________________
2. ______________________________
3. ______________________________
4. ______________________________
5. ______________________________
6. ______________________________
7. ______________________________
8. ______________________________
9. ______________________________
10. ______________________________
11. ______________________________
12. ______________________________
