# Supplementary material for: Perceptions about the relative importance of patient care-related topics: a single institutional survey of its anesthesiologists, nurse anesthetists, and surgeons
Source: BMC Anesthesiol. 2016 Mar 22;16:19. doi: 10.1186/s12871-016-0187-0 (PMC4804473; doi:10.1186/s12871-016-0187-0)
Supplement: Supplementary file 2 — Perioperative Risk Optimization and Management Planning Tool (PROMPT™) Quantitative Clinician Survey. (PDF 73 kb) [file 12871_2016_187_MOESM2_ESM.pdf]

<u>Surveying the Clinical Practice Patterns within the UAB Department of Anesthesiology</u>

Quantitative PROMPT Survey – <u>STUDY SURVEY TWO</u>

<u>Please answer the following questions</u>:

What type of clinician are you? ☐ Anesthesiologist ☐ Surgeon ☐ Nurse Anesthetist

How many years after completing your training have you been in clinical practice? ______ years

How old are you? ______ years

What is your gender? ☐ Female ☐ Male

What is your race? ☐ African-American ☐ Caucasian ☐ Hispanic ☐ Other

A Perioperative Risk Optimization and Management Planning Tool (<u>PROMPT</u>) is a local clinician-designed approach to promoting standardization of care, which accommodates patients' individual differences, respects care providers' clinical acumen, and keeps pace with the rapid growth of medical knowledge.

There are a number of possible topics for the development and implementation of PROMPTs here at UAB.

Please rate HOW IMPORTANT you think <u>each</u> of the following perioperative topics is on a scale of 1 to 10, with 1 being NOT AT ALL IMPORTANT and 10 being EXTREMELY IMPORTANT.

These topics include the categories of Preoperative Testing, Medications and Technologies, Optimization of Co-Morbid Diseases, Perioperative Risk Reduction, and Provision of Comprehensive Care.

A. Preoperative Testing
  1. Laboratory
  2. Cardiovascular
  3. Pulmonary function

B. Medications and Technologies
  1. Beta-blockers
  2. Calcium channel blockers
  3. Diuretics
  4. Angiotensin-converting enzyme inhibitors
  5. Angiotensin receptor blockers
  6. Anticoagulants
  7. Coronary artery stents
  8. Cardiac pacemakers
  9. Implantable cardioverter defibrillators
  10. Insulin pumps
  11. Intrathecal pumps

12. Other implantable devices (vagal nerve stimulators, deep brain stimulators)

C. Optimization of Co-Morbid Diseases

1. Arterial hypertension
2. Coronary artery disease
3. Cardiomyopathy
4. Congestive heart failure
5. Cardiac arrhythmias
6. Diabetes mellitus
7. Cerebrovascular disease
8. Chronic obstructive pulmonary disease
9. Obstructive sleep apnea
10. Pulmonary hypertension
11. Renal insufficiency
12. Cirrhosis and liver failure
13. Anemia
14. Obesity
15. Infection and sepsis
16. Trauma
17. Chronic pain/chronic opioid use
18. Substance abuse
19. Malignant hyperthermia
20. Reported penicillin allergy

D. Perioperative Risk Reduction

1. Cognitive delirium
2. Cognitive dysfunction
3. Nausea and vomiting
4. Deep venous thrombosis
5. Cerebrovascular accident
6. Myocardial injury after non-cardiac surgery (“MINS”)
7. Acute kidney injury and renal failure

E. Provision of Comprehensive Care

1. Choice of anesthetic technique and agents
2. Airway management
3. Intraoperative ventilation
4. Glucose management (not just in diabetes)
5. Nutrition
6. Fluid and electrolyte management
7. Patient-centered blood management
8. Patient medication instruction and compliance
9. Preoperative physical conditioning (“prehabilitation”)
10. Smoking cessation
11. Perioperative opioid sparring strategies (“multimodal analgesia”)

12. Perioperative sedative sparring strategies
13. Preoperative antimicrobial prophylaxis and skin preparation
14. Intraoperative hypothermia prevention (maintaining normothermia)
15. Geriatric anesthetic and analgesic management
